# A NEW ROLE FOR KYNURENINE METABOLISM IN THE AGE-ASSOCIATED DECLINE OF THE BACTERIAL IMMUNE RESPONSE

**DOI:** 10.1093/geroni/igad104.2989

**Published:** 2023-12-21

**Authors:** George Sutphin, Luis Espejo

**Affiliations:** University of Arizona, Tucson, Arizona, United States; University of Arizona, Tucson, Arizona, United States

## Abstract

Tryptophan metabolism through the kynurenine pathway becomes dysregulated during normal aging and is implicated in age-associate disease, including chronic inflammation, atherosclerosis, neurodegeneration, and cancer. Kynurenine pathway enzymes and metabolites influence a range of molecular processes critical to healthy aging, including regulation of inflammatory and immune responses. Kynurenine metabolism is active in immune cells and activated in response to proinflammatory cytokine signaling. We previously determined that elevating physiological levels of the kynurenine pathway metabolite 3-hydroxyanthranilic acid (3HAA) via either direct supplementation or inhibition of the enzyme that degrades 3HAA, 3HAA dioxygenase (HAAO), extends lifespan and delays age-associated health decline in Caenorhabditis elegans. Published work suggests that 3HAA is anti-inflammatory in mammals, for example by reducing the ratio of activated to regulatory T cells and inhibiting pathological activation of macrophages. In recent work, we find that elevating physiological 3HAA can beneficially enhance the immune response of C. elegans to bacterial pathogens during aging. 3HAA is sufficient to kill bacterial and inhibit bacterial growth in culture. When HAAO is inhibited in C. elegans, 3HAA accumulates in lysosome related organelles (LROs) in the intestinal cells, the same subcellular compartment that contains engulfed bacteria. LROs are a cellular repository for both iron and zinc, and we further find that iron chelation or zinc supplementation dramatically enhances the bactericidal properties of 3HAA. Here we present a mechanistic model in which age-dependent accumulation of 3HAA in intestinal LROs combines with zinc to enhance bacterial resistance with age in C. elegans.

